# Supplementary material for: Evolutionarily unique mechanistic framework of clathrin-mediated endocytosis in plants
Source: eLife. 2020 Jan 23;9:e52067. doi: 10.7554/eLife.52067 (PMC7012609; doi:10.7554/eLife.52067)
Supplement: Supplementary file 1. [file elife-52067-supp1.docx]

**Supplementary file, table 1**:

| **Basket-type** | **Degree of invagination** | **No. of CCPs** |
| --- | --- | --- |
| Hexagonal | Dome-shaped | 47 |
|  | Spherical | 16 |
|  | Flat | 2 |
| Pentagonal | Dome-shaped | 15 |
|  | Spherical | 6 |
|  | Flat | 1 |
| Irregular | Dome-shaped | 35 |
|  | Spherical | 25 |
|  | Flat | 3 |
| **Total** | | **151** |

**Supplementary file, table 2**:

| System: Root | | | | | |
| --- | --- | --- | --- | --- | --- |
| EAP | Treatment | Average Lifetime τ (s) | Density ⍴  (# foci/100 µm^2^) | N=number of cells; tracks | Significance |
| CLC2-GFP | Mock | 21.93 ± 2.42 | 87.05 ± 12.47 | 5 cells; 32990 tracks | ns (τ;⍴) |
|  | LatB | 20.66 ± 3.51 | 87.47 ± 3.854 | 4 cells; 25517 tracks |  |
|  | Mock | 24.96 ± 6.33 | 88.76 ± 8.17 | 3 cells; 17901 tracks | ns (τ;⍴) |
|  | Jasp | 23.93 ± 3.00 | 81.61 ± 9.17 | 6 cells; 40227 tracks |  |
| TPLATE-GFP | Mock | 22.64 ± 2.261 | 90.27 ± 10.81 | 5 cells; 30577 tracks | ns (τ;⍴) |
|  | LatB | 25.20 ± 2.80 | 83.53 ± 16.02 | 4 cells; 19259 tracks |  |
| Drp1C-GFP | Mock | 25.67 ± 2.89 | 50.97 ± 8.833 | 6 cells; 27163 tracks | ns (τ;⍴) |
|  | LatB | 26.26 ± 2.14 | 71.91 ± 31.46 | 4 cells; 17923 tracks |  |
|  | Mock | 27.52 ± 2.64 | 68.19 ± 10.95 | 3 cells; 11611 tracks | ns (τ);  Significant (⍴) |
|  | Jasp | 26.46 ± 1.98 | 71.98 ± 11.21 | 6 cells;  17866 tracks |  |
| System: Hypocotyl | | | | | |
| CLC2-GFP | Mock | 10.43 ± 0.43 | 72.60 ± 4.86 | 3 cells; 10883 tracks | ns (τ;⍴) |
|  | LatB | 10.02 ± 0.73 | 66.78 ± 10.49 | 4 cells; 12623 tracks |  |
|  | Mock | 11.37 ± 0.27 | 88.53 ± 2.51 | 3 cells; 11132 tracks | ns (τ;⍴) |
|  | Jasp | 11.07 ± 0.55 | 77.97 ± 10.71 | 3 cells; 7876 tracks |  |
| TPLATE-GFP | Mock | 9.88 ± 0.78 | 97.50 ± 16.98 | 4 cells; 13306 tracks | ns (τ;⍴) |
|  | LatB | 9.13 ± 1.00 | 84.84 ± 11.42 | 3 cells; 12642 tracks |  |
| Drp1C-GFP | Mock | 16.76 ± 0.64 | 81.15 ± 11.60 | 3 cells; 11004 tracks | ns (τ;⍴) |
|  | LatB | 14.57 ± 1.57 | 72.53 ± 6.69 | 4 cells; 14658 tracks |  |
